# Controlled Production of Zearalenone-Glucopyranoside Standards with *Cunninghamella* Strains Using Sulphate-Depleted Media

**DOI:** 10.3390/toxins13060366

**Published:** 2021-05-21

**Authors:** Jeroen Peters, Edward Ash, Arjen Gerssen, Ruud Van Dam, Maurice C. R. Franssen, Michel W. F. Nielen

**Affiliations:** 1 Wageningen Food Safety Research, Wageningen University and Research, Akkermaalsbos 2, 6708 WB Wageningen, The Netherlands; eash@innosieve.com (E.A.); arjen.gerssen@wur.nl (A.G.); ruud.vandam@wur.nl (R.V.D.); michel.nielen@wur.nl (M.W.F.N.); 2 Innosieve Diagnostics BV, Nieuwe Kanaal 7A, 6709 PA Wageningen, The Netherlands; 3 Laboratory of Organic Chemistry, Wageningen University, Stippeneng 4, 6708 WE Wageningen, The Netherlands; maurice.franssen@wur.nl

**Keywords:** modified mycotoxins, zearalenone, zearalanone, β-zearalenol, conjugated, masked, fungi, *Cunninghamella*, biotransformation

## Abstract

In recent years, conjugated mycotoxins have gained increasing interest in food safety, as their hydrolysis in human and animal intestines leads to an increase in toxicity. For the production of zearalenone (ZEN) glycosides reference standards, we applied *Cunninghamella* *elegans* and *Cunninghamella echinulata* fungal strains. A sulphate-depleted medium was designed for the preferred production of ZEN glycosides. Both *Cunninghamella* strains were able to produce zearalenone-14-β-D-glucopyranoside (Z14G), zearalenone-16-β-D-glucopyranoside (Z16G) and zearalenone-14-sulphate (Z14S). In a rich medium, *Cunninghamella* *elegans* preferably produced Z14S, while *Cunninghamella* *echinulata* preferably produced Z14G. In the sulphate-depleted medium a dramatic change was observed for *Cunninghamella* *elegans*, showing preferred production of Z14G and Z16G. From 2 mg of ZEN in sulphate-depleted medium, 1.94 mg of Z14G and 0.45 mg of Z16G were produced. Following preparative Liquid Chromatography-Mass Spectrometry (LC-MS) purification, both fractions were submitted to ^1^H and ^13^C NMR and High-Resolution Mass Spectrometry (HRMS). These analyses confirmed that the purified fractions were indeed Z14G and Z16G. In conclusion, the presented research shows that a single *Cunninghamella* strain can be an effective and efficient tool for the controlled biotransformation of ZEN glycosides and other ZEN metabolites. Additionally, the biotransformation method was extended to zearalanone, β-zearalenol and other mycotoxins.

## 1. Introduction

Zearalenone (ZEN) is a non-steroidal estrogenic mycotoxin produced by *Fusarium* spp. It occurs in grain commodities, and it can cause reproductive disorders in farm animals and lead to hypoestrogenic syndromes in humans [1]. Because of its toxicity, the European Commission established a tolerable daily intake (TDI) for zearalenone of 0.25 μg/kg of body weight [2]. In 2016, the CONTAM panel of EFSA expanded this TDI to a group health-based guidance TDI value of 0.25 µg per kg of body weight for ZEN and all of its phase I and phase II metabolites. Additionally, potency factors relating to the estrogenic activity of the metabolites were assigned to all the ZEN metabolites [3]. The main occurring ZEN metabolites include α-zearalenol (α-ZEL), β-zearalenol (β-ZEL), zearalanone (ZAN), α-zearalanol (α-ZAL) and β-zearalanol (β-ZAL) [4,5]. Conjugated mycotoxins, often referred to as masked mycotoxins, are biologically modified phase II metabolites produced by plant biotransformations as a detoxification process [6]. Well-known ZEN plant conjugates are zearalenone-14-β-D-glucopyranoside (Z14G), zearalenone-16-β-D-glucopyranoside (Z16G), α-zearalenol-14-β-D-glucopyranoside (α-ZELG), β-zearalenol-14-β-D-glucopyranoside (β-ZELG) and zearalenone-14-sulphate (Z14S) [7,8]. The occurrence of Z14G in wheat was reported by Schneweis et al. [9] when they analyzed 24 Bavarian wheat samples. In total, 10 samples contained Z14G with concentrations ranging from 17 to 104 µg/kg. In their survey of cereal-based foods, De Boevre et al. [10] found the conjugated mycotoxins Z14G, Z14S, α-ZELG and β-ZELG with maximum concentrations of, respectively, 369, 45, 192 and 206 µg/kg. Streit et al. [11] analyzed 139 feed samples, of which 49% contained Z14S. Nathanail et al. [12] analyzed different commodities of Finnish cereal grains and detected Z14G, Z16G, α-ZELG, β-ZELG and Z14S in oats, with the highest concentrations being, respectively, 9.6, 15.1, 5.1, 0.7 and 220 µg/kg. In processed food, Peters et al. [13] detected the presence of Z14S in beers, with concentrations ranging from 0.5 to 0.7 µg/L. Borzekowkski et al. [14] showed that some tempeh products, acquired from Indonesian markets, contained ZEN, α-ZEL and Z14S.

Conjugated mycotoxins can be hydrolyzed into their free forms leading to increased toxicity [6]. Already in 1990, Gareis et al. [15] showed that, when Z14G was fed to pigs, only ZEN and α-ZEL were found back in the urine and feces, indicating hydrolysis of Z14G. Additional research indicated the hydrolysis of Z14S, Z14G and Z16G when fed to pigs [16,17]. Dellaflora et al. [18] showed that Z14G was hydrolyzed to ZEN in bovine blood, while Versilovskis et al. [19] discovered that Z14G fed to rats was successfully hydrolyzed to ZEN. Kovalsky et al. [20] showed that Z16G was hydrolyzed to ZEN using human fecal slurry. These experiments indicate that the presence of these conjugated forms of ZEN lead to additional toxicity and therefore should be detected along with the unconjugated toxins.

To be able to monitor the presence of conjugated mycotoxin forms in food and feed products, enzymatic deconjugation methods or available reference standards are essential. Biotransformation of ZEN with plants and microorganisms has been successfully applied to produce secondary metabolites. Berthiller et al. [21] spiked *Arabidopsis thaliana* plant seedlings with ZEN and found 17 different ZEN conjugates, including glucosides. Next, the responsible *Arabidopsis thaliana* UDP-glycosyl transferase gene was expressed in *Saccharomyces cerevisiae,* and this was used for the direct biotransformation of ZEN yielding Z14G. Part of the produced Z14G was then efficiently chemically modified to α-ZELG and β-ZELG [22,23]. Using a cloned UDP-glycosyl transferase gene from barley, expressed in yeast, Kovalsky et al. [20] were able to produce Z14G and Z16G. Alternatively, root and leaf cultures, made from two durum wheat varieties, selectively produced a wide variety of putative conjugated ZEN metabolites, including malonyl, sulphate, glucoside, maltoside and other di-glycoside forms [24,25].

There is a wide variety of fungal species and, among them, species with unique biochemical pathways. With these biochemical pathways, they are able to produce a wide variety of phase I and phase II metabolites. This ranges from important pharmaceuticals (e.g., antibiotics) to natural toxins (e.g., mycotoxins) [26,27]. These biochemical pathways are also an effective tool to metabolize chemical compounds that are administered to the fungus. Coupling of sugars, or sulphates, to the chemical compound will increase its polarity and makes it easier for the fungus to secrete the conjugated compound into the culture medium [28]. *Cunninghamella* and *Rhizopus* are families of filamentous fungi occurring in soil and plants and are well studied in in vitro biotransformation models. Some observed biotransformations include hydroxylation, glycosylation, oxidation, demethylation, sulfoxidation and epoxidation [29,30]. These fungi have been successfully applied to a wide range of compounds. Applying several strains of the *Rhizopus* family, Brodell et al. [31] and Borzekowski et al. [32] showed that these strains were able to successfully bio-transform ZEN into Z14S, Z14G, Z16G and α-zearalenol-sulfate, and that each had its own preferred pathway as shown by the produced metabolites. El-Sharkawy et al. [33,34] applied liquid cultures of the *Cunninghamella* *bainieri* and *Thamnidium* *elegans* strains to metabolize ZEN. Besides α-ZEL, β-ZEL and β-zearalanol (β-ZAL), the metabolites Z14S and Z14G were produced.

Aiming for the efficient and selective production of ZEN glycosides, two *Cunninghamella* strains, namely *Cunninghamella* *echinulata* var. *elegans (C. elegans)* and *Cunninghamella echinulata* var. *echinulata (C. echinulata),* were ordered based on their ability to glycosylate compounds as reported previously [33,35,36]. With the specific aim of steering the biotransformations to the preferred target glucose-conjugated ZEN metabolites, we applied sulphate-depleted media to avoid excess sulphate metabolite production. Even though comprehensive optimization studies were not undertaken yet, besides prolonged exposure of the fungi to ZEN, we were able to effectively produce the biologically modified [37] Z14G and Z16G. The successful controlled production of ZEN-glycosides was confirmed by ^1^H and ^13^C nuclear magnetic resonance (NMR), both 1D and 2D, and high-resolution mass spectrometry (HRMS) analysis. In addition, the functionality of the controlled glycosylation-directed biotransformation by the selected *Cunninghamella* strains was also tested for ZAN, β-ZEL and a range of other common mycotoxins.

## 2. Results and Discussion

### 2.1. Small-Scale Biotransformation of ZEN

**Figure 1:**
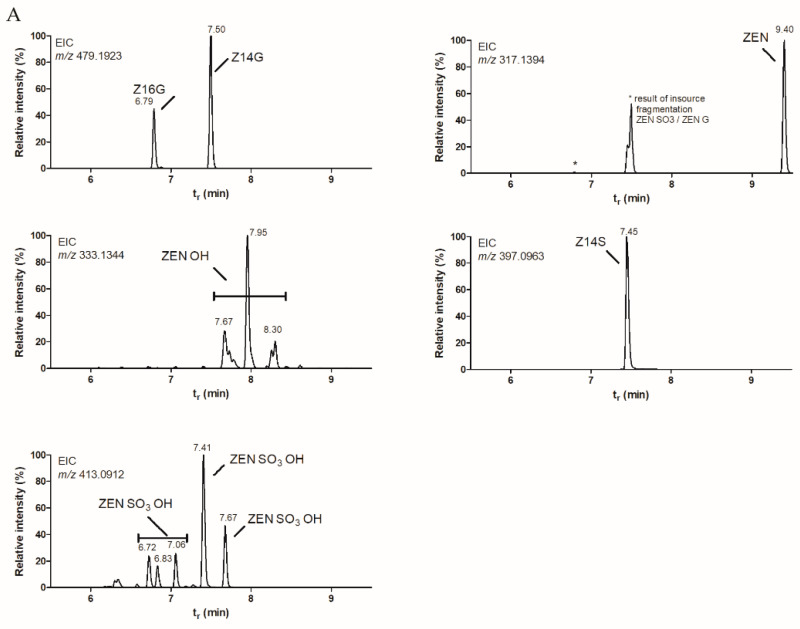

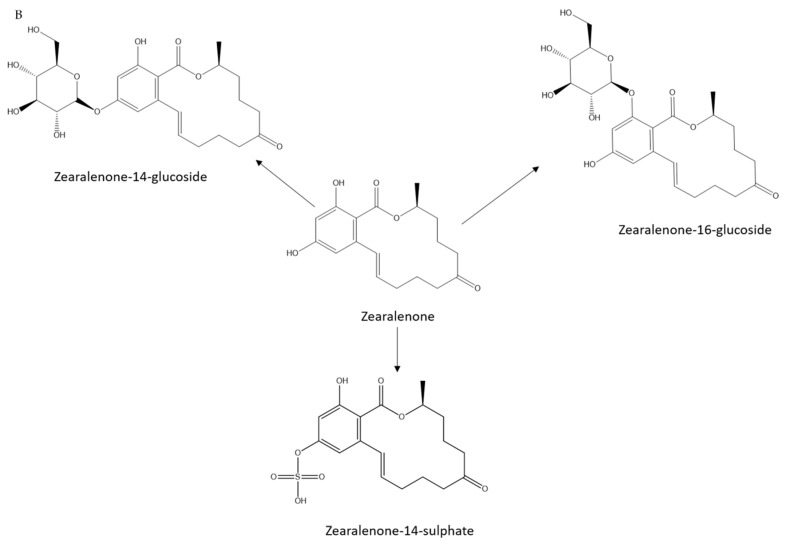
(**A**) Formation of various ZEN metabolites by *Cunninghamella elegans*. Tentative identified with LC-HRMS based on the exact mass of both the precursor ions and specific fragments (*m/z* 131.0505 and 175.0403). Extracted ion chromatograms of the precursor ions are shown. (**B**) Chemical structures of ZEN and the main phase II metabolites produced by *Cunninghamella elegans*.

For initial experiments, we adopted a protocol applied in quercetin biotransformation [35]. Using non-optimized small-scale cultures of *C. elegans* in potato dextrose broth (PDB) medium, 25 ug/mL ZEN was added to a 3-day culture and incubated for 144 h. Supernatant samples were analyzed using HRMS. This experiment revealed that *C. elegans* was also capable of metabolizing ZEN. Metabolites formed included the desired Z14G and Z16G conjugates, as well as Z14S, but the parent compound ZEN was not completely metabolized (Figure 1A,B).

**Figure 2:**
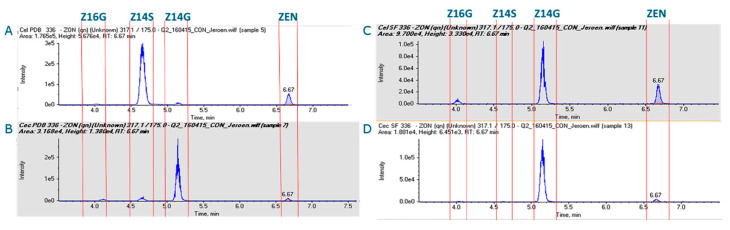
LC-MS/MS chromatograms (*m/z* 317.1→175.0) for the production of Z14G, Z16G and Z14S metabolites by *Cunninghamella elegans* in PDB medium (**A**) and synthetic depletion medium (**C**) and *Cunninghamella echinulata* in PDB medium (**B**) and synthetic depletion medium (**D**).

Next, a dedicated LC-MS/MS method was set up using reference and purified standards of ZEN, Z14G, Z16G and Z14S. The retention times and MS/MS characteristics of each molecule were determined. This allowed the selective quantification of each target conjugate during analysis. Because of identical product ion masses, LC retention time was crucial for the identification of the Z14G and Z16G metabolites. Separation of Z14G and Z14S, not realized in the initial HRMS runs, was satisfactory. With the biotransformation incubation times adjusted to 336 h, and the ZEN concentration adjusted to 5 µg/mL, biotransformation was more optimal and *C. elegans* seemed to remain viable, as visually observed by continued growth of the culture. Next, fungal cultures of *C. elegans* and *C. echinulata* were fortified with ZEN in PDB and in the modified Czapek-Dox (MCD) sulphate-depleted media. With the depletion of sulphates in the MCD medium, we intended to direct the biotransformation towards the glycosylated metabolites while minimizing the Z14S production. Figure 2 shows the LC-MS/MS chromatograms of the respective supernatants of those biotransformations.

As each conjugate contains ZEN, the *m/z* 175.0 product ions will appear in the chromatograms at their respective retention times. Focusing on the rich PDB medium, *C. elegans* predominantly produced Z14S, with only minor traces of Z14G and Z16G. *C. echinulata* predominantly converted ZEN into Z14G, whilst also producing small amounts of Z16G and Z14S. The *C. elegans* and *C. echinulata* cultures that were washed and then transferred to the minimal sulphate MCD media showed interesting differences in the biotransformation of ZEN when compared to the PDB cultures. Most prominent was the shift from the production of Z14S by *C. elegans* in PDB to the production of Z14G and Z16G. Moreover, no significant amount of Z14S was produced. The *C. echinulata* strain mainly produced Z14G in the MCD medium. This shift was less dramatic when compared to *C. elegans*. Based on these results we decided to continue the production of ZEN glycosides using the *C. elegans* strain and the MCD medium.

### 2.2. Upscaled Production of ZEN-Glycosides

To increase the production, *C. elegans* cultures were upscaled 40 times, where 2 mg of ZEN was introduced from a stock solution into 400 mL of MCD in a 2 L flask. For efficiency comparison, another 2 L flask containing 400 mL of PDB was also fortified with 2 mg of ZEN. After 2 weeks’ incubation, supernatants were analyzed by LC-MS/MS using standard curves in MCD and PDB media for calibration. Based on these standard curves it was calculated that from 2 mg ZEN, 1.96 mg of Z14G and 0.45 mg Z16G were produced in MCD medium. On a molar ratio this meant that 65% of ZEN was metabolized to Z14G, while 14% ZEN was metabolized to Z16G. In PDB medium, those efficiencies were only 3% for the production of Z14G and 2% for Z16G (Table 1).

The calculated recoveries only refer to the supernatants of the cultures, as extraction from mycelia was not considered. The large-scale experiment was then repeated in two 2 L Erlenmeyer flasks, with each 2 mg of ZEN spiked in 400 mL of MCD in order to generate sufficient metabolites for the subsequent steps. While the efficiency of Z14G production was comparable to that in the research of Borzekowski et al. [32], the Z16G production was a bit less efficient. However, in the current research, only a single fungal strain was sufficient to selectively produce two different ZEN-glycosides. This single *C. elegans* strain could also predominantly produce Z14S by using a rich growth medium.

### 2.3. NMR Analysis of Purified Z14G and Z16G Fractions

Prior to NMR analysis, the produced ZEN glycosides were purified by preparative LC-MS after liquid–liquid extraction by an external propriety method. Purified Z14G and Z16G fractions were collected over several runs and subsequently pooled and freeze-dried. Next, the freeze-dried Z14G and Z16G fractions were each dissolved in 2 mL of 50:50 *v*/*v* ACN/H_2_O. All NMR spectra and corresponding data are deposited in the Supplementary Materials. The ZEN chemical structure numbering is shown in Figure S1. The ^1^H, ^13^C and DEPT NMR spectra of ZEN are shown in Figures S2–S4. 2D-NMR techniques provided the identity of each peak (Figure S5). The ^1^H and ^13^C NMR spectra for Z14G are shown in Figures S6 and S7. The ^13^C spectrum was similar to ZEN, except for the addition of the glucose peaks between 100 and 60 ppm, surrounding the peak of C2 at 73 ppm. Due to the similarities in the spectra between ZEN and Z14G, assigning the peaks was easy, and the clear coupling between C14 and C19 seen on the HMBC confirmed the compound as Z14G, see Figures S8 and S9. Just like in ZEN, C14 and C16 could be identified by whether they only coupled to the hydrogen at C15, or also to the one at C13. For Z16G, the ^1^H NMR spectrum (Figure S10) had more overlapping peaks than those for ZEN and Z14G, and the ^13^C NMR spectrum had a wavy baseline due to the small amount of material (Figure S11). The peaks for the alkene hydrogens were different from Z14G, both having a chemical shift near 6.2 ppm, instead of C11 having a much higher chemical shift as in ZEN and Z14G. The peak in the ^1^H NMR for the hydrogen on the anomeric glucose carbon, C19, was hidden under the solvent peak at 3.3 ppm. A small shoulder can be seen to the left of the peak, as shown in Figure S12, and the identity of this as a compound peak was shown by the HSQC coupling between the C19 peak (at 102.8 ppm) on the ^13^C NMR spectrum (Figure S11) and the shoulder (Figure S13). The key HMBC and COSY couplings are shown in Figures S13 and S14. Further data specification can be found in Tables S1–S6. Additionally, the purified standards were also submitted to LC-HRMS to obtain high-resolution MS and fragmentation spectra. These spectra are shown in Figures S15 and S16 of the Supplementary Information. Besides the deprotonated molecular ion, both Z14G and Z16G produced a formic acid adduct. For the fragmentation spectra, these formic acid adducts were isolated and fragmented. From the MS spectra it became clear that Z14G also showed intense radical anions. The obtained *m/z* values for all deprotonated ions, adducts and fragments were within a 5 ppm mass error.

### 2.4. Feasibility of Biotransformation for Other ZEN Metabolites

**Figure 3:**
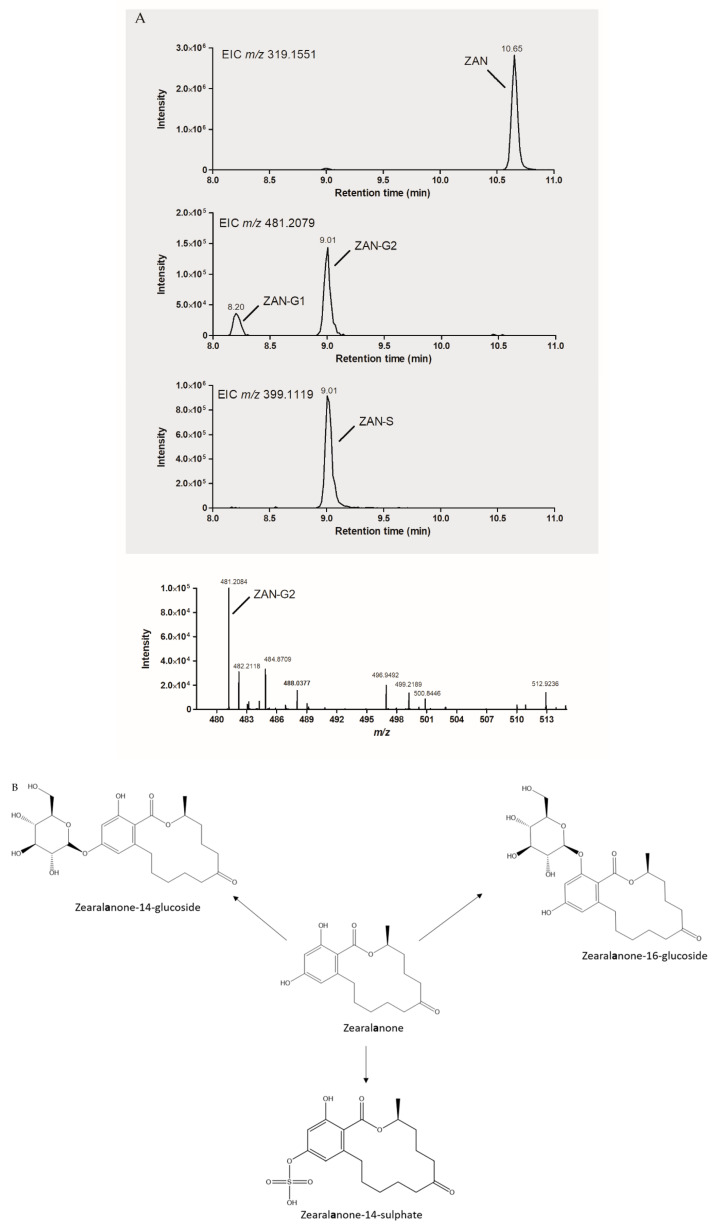
(**A**) LC-HRMS analysis of ZAN biotransformation by Cunninghamella *elegans* in PDB. (**B**) Chemical structures of ZAN and the putative sulphate and glucose phase II metabolites produced by Cunninghamella *elegans*.

**Figure 4:**
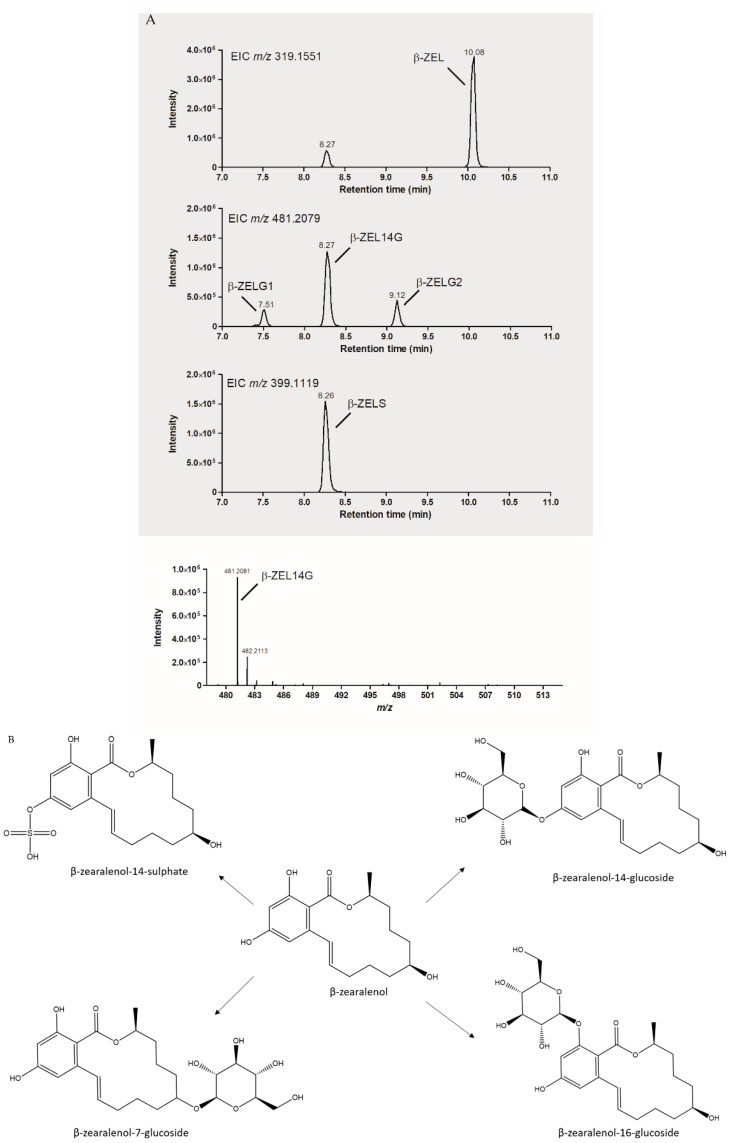
(**A**)**.** LC-HRMS analysis of β-ZEL biotransformation by Cunninghamella *echinulata* in PDB. (**B**) Chemical structures of β-ZEL and the main phase II metabolites (and putative metabolites) produced by Cunninghamella *elegans*.

In a short feasibility study, ZAN and β-ZEL were spiked independently to both Cunninghamella cultures, in PDB and MCD media, at a concentration of 5 µg/mL. The fortified cultures were incubated for 2 weeks, as described previously, and the supernatants were analyzed using a standard HRMS approach with ZAN, β-ZEL and β-ZELG as available reference standards. Tables S7 and S8 show the ions of the putative ZAN and β-ZEL metabolites formed based on HRMS analysis. Results indicate that ZAN was metabolized to zearalanone-sulphate and two forms of zearalanone-glucoside (Figure 3A,B), while β-ZEL showed three possible glucoside metabolites and a sulphate metabolite (Figure 4A,B).

This suggests that besides β-ZELG (standard was available, glucose moiety at position 14), β-zearalenol-16-glucoside and β-zearalenol-7-glucoside also were formed. In both biotransformations, only one putative sulphate conjugated metabolite was observed. Based on previous research [32], it is presumable that the ZAN and β-ZEL metabolites have the sulphate moiety attached on position 14. However, upscaled production of all metabolites produced, followed by NMR analysis, is necessary to confirm this.

### 2.5. Feasibility of Biotransformation for Entirely Different Mycotoxins

In additional exploratory experiments, using the same approach as for ZEN and its metabolites, the Cunninghamella biotransformation strategy was applied to the mycotoxins deoxynivalenol (DON), aflatoxin B_1_ (AFB_1_), fumonisin B_1_ (FB_1_), T2-toxin (T-2) and ochratoxin A (OTA). For DON, AFB_1_ and FB_1_ no obvious biotransformations were observed at the conditions previously used for ZEN. No predicted conjugates (e.g., hydroxy, sulphate, glucose) were found in HRMS analysis; moreover, no decrease in mass balance was observed versus the initial concentrations of the fortified mycotoxins. McCormick et al. [38] previously reported the formation of T2-glycosides by certain yeast strains, but both Cunninghamella strains applied in our research were not able to produce these target glycosides. However, upon addition of T-2, we did observe two other biotransformations: to HT2-toxin and to hydroxy-T2 Toxin. The degradation of OTA by Rhizopus strains was previously reported [39]. Formation of OTA-glycosides by plant cell suspension cultures was also previously reported [40,41]. *C. elegans* very effectively transformed OTA to hydroxy-OTA within 96 h after fortification, but no phase II metabolites were observed, probably because the new OH group at the lactone ring is sterically hindered while the phenolic OH moiety is involved in hydrogen bonds with the adjacent carbonyl groups.

## 3. Conclusions

The implementation of sulphate-depleted media in Cunninghamella-based biotransformation of ZEN proved to be a successful method for steering the reaction towards the preferred abundant production of ZEN-glycosides. Although in general the transformation efficiencies were satisfying, ZEN was not fully metabolized by the fungal culture and was still present in the supernatant. To further optimize future production, the implementation of larger culture volumes in dedicated bioprocessors, tweaking temperatures and especially aeration [42], while fortifying at lower ZEN concentrations may lead to more optimal production of ZEN glycosides. Additionally, the fungal matter could also be extracted to further increase biotransformation efficiency. Besides the effective glycoside production, it is at the same time an effective tool to produce the Z14S metabolite when using the standard PDB growth medium in combination with *C. elegans*. The HRMS run of a sub-optimal biotransformation (Figure 1A) revealed several phase I and phase II ZEN metabolites, including ZEN-hydroxy compounds. These most likely are α-ZEL and β-ZEL. Subsequent phase II metabolism may turn these into α-ZELG and β-ZELG, as shown by the formation of three glucose metabolites from β-ZEL in preliminary experiments. However, based on the mass balance, it seems that Z14S, Z14G and to a lesser extent Z16G were the conjugates that were preferably produced under the conditions investigated. Besides transformation efficiency optimization, future research could also focus on the implementation of a wider range of Cunninghamella strains and determine their preferred pathways in rich and selective growth media.

The developed method is easy implementable, does not need extensive microbiological experience and does not have complex work schemes. It may be a useful tool for production of metabolites in case novel or emerging toxin metabolites are discovered and reference standards are not commercially or scientifically available.

## 4. Materials and Methods

### 4.1. Instrumentation

Fungal culture streaks were grown in a temperature-controlled incubator (Van Tol laboratorium techniek, Kerkdriel, The Netherlands). All sizes of liquid fungal cultures were grown in an Innova 44 rotary shaker (New Brunswick Scientific, Edison, USA) and centrifuged in an Eppendorf 5810R centrifuge (Eppendorf, Nijmegen, The Netherlands) equipped with an A-4-62 swinging bucket rotor. The formation of ZEN conjugates was monitored on an AB Sciex (Nieuwerkerk a/d IJssel, The Netherlands) QTRAP 6500 tandem mass spectrometer (MS/MS) equipped with an electrospray ionization (ESI) source, operated in negative ion multiple reaction monitoring (MRM) mode. The MS system was coupled to a Shimadzu (‘s Hertogenbosch, The Netherlands) Prominence Liquid Chromatography (LC) system, equipped with a Restek (Interscience, Breda, The Netherlands) Ultra Aqueous C18 (100×2.1 mm) column. Integration of reconstructed MRM chromatograms was done with MultiQuant V2.0 software using the Signal Finder integration algorithm (AB Sciex). Produced ZEN-glycosides were purified according to a propriety method of the Federal Institute for Materials Research and Testing (BAM, Berlin, Germany). NMR measurements for ZEN and Z14G were performed using a Bruker Avance III 400MHz NMR spectrometer, recorded by Topspin software at 25 °C against internal standard TMS at 0.00 ppm. For Z16G, the ^1^H and ^13^C NMR spectra were recorded at a probe temperature of 300 K on a Bruker Avance-III-600 spectrometer, equipped with a cryo-probe located at MAGNEFY (MAGNEtic resonance research FacilitY, Wageningen, The Netherlands). 1D and 2D COSY, HMBC and HMQC spectra were acquired using standard pulse sequences delivered by Bruker. For the HRMS experiments of the produced ZEN-glycosides a Q-Exactive Orbitrap mass spectrometer equipped with a HESI-II electrospray source was used (Thermo Scientific, San Jose, CA, USA). The HRMS system was coupled to an Ultimate 3000 UHPLC LC system (Thermo Scientific, San Jose, CA, USA) equipped with a 100 × 3 mm ID, 3 μm Atlantis T3 analytical column (Waters, Milford, MA, USA). Extracted ion chromatograms were constructed with the Thermo Scientific Xcalibur software. GraphPad Prism 4 was used for building graphics where possible.

### 4.2. Materials

Fungal strains *Cunninghamella* *echinulata* var. *elegans* (ATCC 9245, deposited name *Cunninghamella* *blakesleeana* Lendner) and *Cunninghamella* *echinulata* var. *echinulata* (ATCC 9244, deposited name *Cunninghamella* *bainieri* Naumov) were ordered from LGC (Wesel, Germany) and BCCM (Brussels, Belgium). Potato dextrose broth (PDB), potato dextrose agar (PDA) and 2 L Erlenmeyer flasks for growing large-scale cultures were purchased from Sigma-Aldrich (Zwijndrecht, The Netherlands). Fungi were plated in Petri dishes, and small-scale cultures were grown in disposable 50 mL tubes, both from Greiner (Alphen a/d Rijn, The Netherlands). ZEN, ZAN and β-ZEL in solid form were purchased from Fermentek (Jerusalem, Israel). Z14G and Z16G standards were kindly provided by Dr Franz Berthiller (IFA Tulln, Austria), while the Z14S standard was kindly provided by Dr Matthias Koch (BAM, Germany). Syringeless filter devices (Mini-UniPrep, PTFE) were purchased from GE Healthcare (Rotterdam, The Netherlands). Acetonitrile (ACN) and methanol (MeOH) were purchased from Biosolve (Valkenswaard, The Netherlands), acetic acid, formic acid and ammonium formate from Merck (Amsterdam, The Netherlands) and ammonium acetate from Fluka Analytical (Steinheim, Germany). For the HRMS experiments, ACN, MeOH and water, all of UHPLC-MS purity grade, were purchased from Merck. All other chemicals were purchased either from VWR International (Amsterdam, The Netherlands) or Sigma-Aldrich (Zwijndrecht, The Netherlands).

### 4.3. Fungal Starter Cultures

For small-scale cultures, fungal mycelia were transferred from PDA plates to 50 mL tubes, containing 10 mL of PDB, by an inoculation loop. After inoculation, the tubes were closed and vortexed vigorously. For large-scale cultures, fungal mycelia were first transferred from PDA plates into a 50 mL tube containing 10 mL of PDB. After inoculation, the tube was closed and vortexed vigorously. Next, 5 mL of fungal suspension was transferred to 400 mL of PDB in a 2 L Erlenmeyer flask. The tubes and flasks were then placed in a rotary shaker and incubated at 27 °C while shaking at 200 RPM for a total of 3 days.

### 4.4. Biotransformation of ZEN in Liquid Fungal Cultures

After 3 days of stress-free growth, ZEN at a final concentration of 5 µg/mL was added to the fungal cultures in PDB using a stock solution of 1 mg/mL of ZEN (prepared by dissolving 5 mg of solid ZEN in 5 mL of MeOH). After the addition of ZEN, the fungal cultures were further incubated with a final optimal biotransformation time of 2 weeks. For steering the biotransformation towards the preferable production of ZEN-glucosides, a new growth medium was developed. The new medium was based on Czapek–Dox medium, a growth medium based on inorganic salts, sugar and water. All sulphate-based salts were omitted from the modified Czapek–Dox medium (MCD) and were not replaced by other salts. The adjusted medium then consisted of 30% dextrose, 2% NaNO_3_, 0.5% KCl and 1% K_2_HPO_4_ in double-distilled water, set at pH 7.3. The adjusted biotransformation procedure was as follows. After 3 days of stress-free growth, fungal cultures were centrifuged at 3000× *g* for 10 min, and the supernatant (PDB) was removed. The fungal mycelia were then washed 1 time with double-distilled water and again centrifuged at 3000× *g* for 10 min. The fungal mycelia were reconstituted in the original culture volume using MCD medium and fortified at 5 µg/mL ZEN. These fortified cultures were then further incubated for 2 weeks at 27 °C in a rotary shaker set to 200 RPM.

### 4.5. LC-MS/MS Analysis of Produced Conjugates

An LC-MS/MS method was developed based on available ZEN, Z14G, Z16G and Z14S reference standards (Figure S17). Before LC-MS/MS measurements, the fungal cultures were centrifuged at 3000× *g* for 10 min, and the supernatant was collected. The supernatant was filtered through a syringeless filter device, and 5 µL was injected into the LC system, applying a flow of 0.40 mL/min and a column temperature of 35 °C. A gradient was applied (Table S9) using running buffer A that consisted of 5 mM ammonium acetate + 0.1% acetic acid in water and running buffer B that consisted of 5 mM ammonium acetate + 0.1% acetic acid in MeOH/H_2_O *v*/*v* 95/5. Detection of ZEN, and its main formed phase II metabolites, was performed in a negative MRM mode according to the settings shown in Table S10. Concentrations of the produced metabolites were calculated by creating dose–response curves (from 10 ng/mL to 1 µg/mL) of the available standards in the fungal growth medium.

### 4.6. NMR Analysis of Produced Conjugates

Initially, 10 mg of ZEN was dissolved in deuterated methanol (MeOD), and ^1^H, ^13^C, ^13^C-DEPT, HSQC, COSY and HMBC analyses were carried out to identify the various peaks. Then, the produced conjugates were also dissolved in MeOD, and a ^1^H NMR analysis was carried out to determine if enough conjugated ZEN metabolites were available. Next, ^1^H, ^13^C, ^13^C-DEPT, HSQC, COSY and HMBC analyses were carried out on the 400 MHz NMR spectrometer. Since the amount of Z16G was very low (<0.5 mg), a 600 MHz NMR spectrometer fitted with a cryoprobe was used for ^1^H, ^13^C, HSQC, COSY and HMBC analyses.

### 4.7. LC-HRMS Analysis of the ZEN Metabolites by Cunninghamella and Collection of HRMS Spectra of the Purified ZEN Glycosides

For the separation of ZEN metabolites in the fungal extract, after filtration, 10 µL was injected on the analytical column, which was kept at 40 °C. The ZEN metabolites were separated with a gradient elution (Table S11) using running buffer A that consisted of 2 mM ammonium formate and 0.002 *v*/*v*% formic acid in water and running buffer B that consisted of 2 mM ammonium formate and 0.002 *v*/*v* % formic acid in acetonitrile/water *v*/*v* 90/10. The flowrate was kept constant at 0.3 mL/min. The HRMS operated in negative ionization mode, and the capillary temperature was set at 250 °C with a spray voltage of 3.5 kV. A full scan event followed by an all ion-fragmentation scan event was applied. The full scan data were recorded with a *m/z* range of 120–1200 with a resolution setting of 35,000, the automatic gain control (AGC) set at 5e5 and the maximum injection time (IT) set at 200 ms. For the all ion fragmentation, a resolution setting of 17,500, an AGC of 3e6 and an IT of 200 ms and a stepped normalized collision energy (NCE) of 40 and 60 were used. The MS/MS spectra were obtained by infusing the purified standard in diluted 2 mM ammonium formate and 0.002 *v*/*v* % formic acid in MeOH/water *v*/*v* 95/5 at a flowrate of 10 µL/min. Spectra were obtained at a resolution setting of 70,000. For the MS/MS fragmentation the formic acid adduct [M+FA-H]^−^ *m/z* 525.19720 +/− 2 Da was isolated and fragmented with 50 NCE.

## Figures and Tables

**Table 1:** Calculated efficiencies for ZEN-glycosides production from 2 mg of ZEN in potato dextrose and sulphate-depleted growth medium (MCD) (n.d. = not determined).

		Potato Dextrose Broth Medium (PDB)			Sulphate-Depleted Medium (MCD)		
Compound	MW	Amount (µg)	Amount (µM)	Conversion (%)	Amount (µg)	Amount (µM)	Conversion (%)
ZEN	318.4	2000	6.28	n.d.	2000	6.28	n.d.
Z14G	480.1	80	0.167	3	1960	4.08	65
Z16G	480.1	56	0.117	2	440	0.92	15

## Data Availability

Not applicable.

## Supplementary Materials

The following are available online at https://www.mdpi.com/article/10.3390/toxins13060366/s1, Figure S1: Numbering of atoms for ZEN and its glucosides, Figure S2: ^1^H-NMR spectrum of ZEN, Figure S3: ^13^C-NMR spectrum of ZEN, Figure S4: DEPT spectrum of ZEN, Figure S5: The most important 2D-coupling signals in ZEN, Figure S6: ^1^H-NMR spectrum of Z14G, Figure S7: ^13^C-NMR spectrum of Z14G, Figure S8: Key HMBC coupling between C14 and C19 in Z14G, Figure S9: Key HMBC and COSY interactions in Z14G, Figure S10: ^1^H-NMR spectrum of Z16G, Figure S11: ^13^C NMR spectrum for Z16G, Figure S12: Z16G ^1^H NMR, showing the shoulder on the MeOD peak indicating the proton on the anomeric C19 which couples to C16, Figure S13: Key HMBC coupling between C16 and C19 in Z16G, Figure S14: Key HMBC and COSY interactions in Z16G, Figure S15: HRMS fragmentation spectra of Z14G, Figure S16: HRMS fragmentation spectra of Z16G, Figure S17: Applied ZEN reference standards in the developed LC-MS/MS method, Table S1: ^1^H NMR data of ZEN, Table S2: ^13^C NMR data of ZEN, Table S3: ^1^H-NMR data of Z14G, Table S4: ^13^C-NMR data of Z14G, Table S5: ^1^H-NMR data of Z16G, Table S6: ^13^C-NMR data of Z16G, Table S7: Calculated and HRMS established ions *(m/z)* of ZAN biotransformation metabolites, Table S8: Calculated and HRMS established ions *(m/z)* of β-ZEL biotransformation metabolites, Table S9: LC gradient (LC-MS/MS), Table S10: Mycotoxin specific MS/MS settings for the negative ionization mode and Table S11: LC gradient (HRMS).
