# Supplementary material for: Controlled Production of Zearalenone-Glucopyranoside Standards with Cunninghamella Strains Using Sulphate-Depleted Media
Source: Toxins (Basel). 2021 May 21;13(6):366. doi: 10.3390/toxins13060366 (PMC8224279; doi:10.3390/toxins13060366)
Supplement: Supplementary file 1 [file toxins-13-00366-s001.zip › toxins-1226185-supplementary for final JP.pdf]

Supplementary Materials: Controlled Production of Zearalenone-Glucopyranoside Standards with *Cunninghamella* Strains Using Sulphate-Depleted Media

Jeroen Peters, Edward Ash, Arjen Gerssen, Ruud Van Dam, Maurice C. R. Franssen and Michel W. F. Nielen

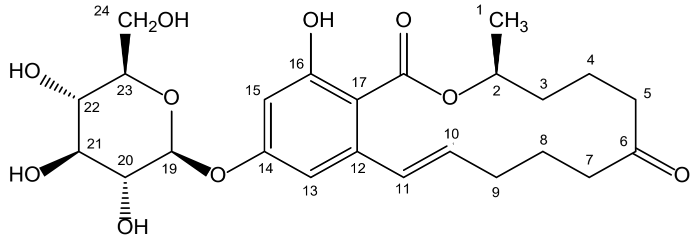

Figure S1. Numbering of atoms for ZEN and its glucosides.

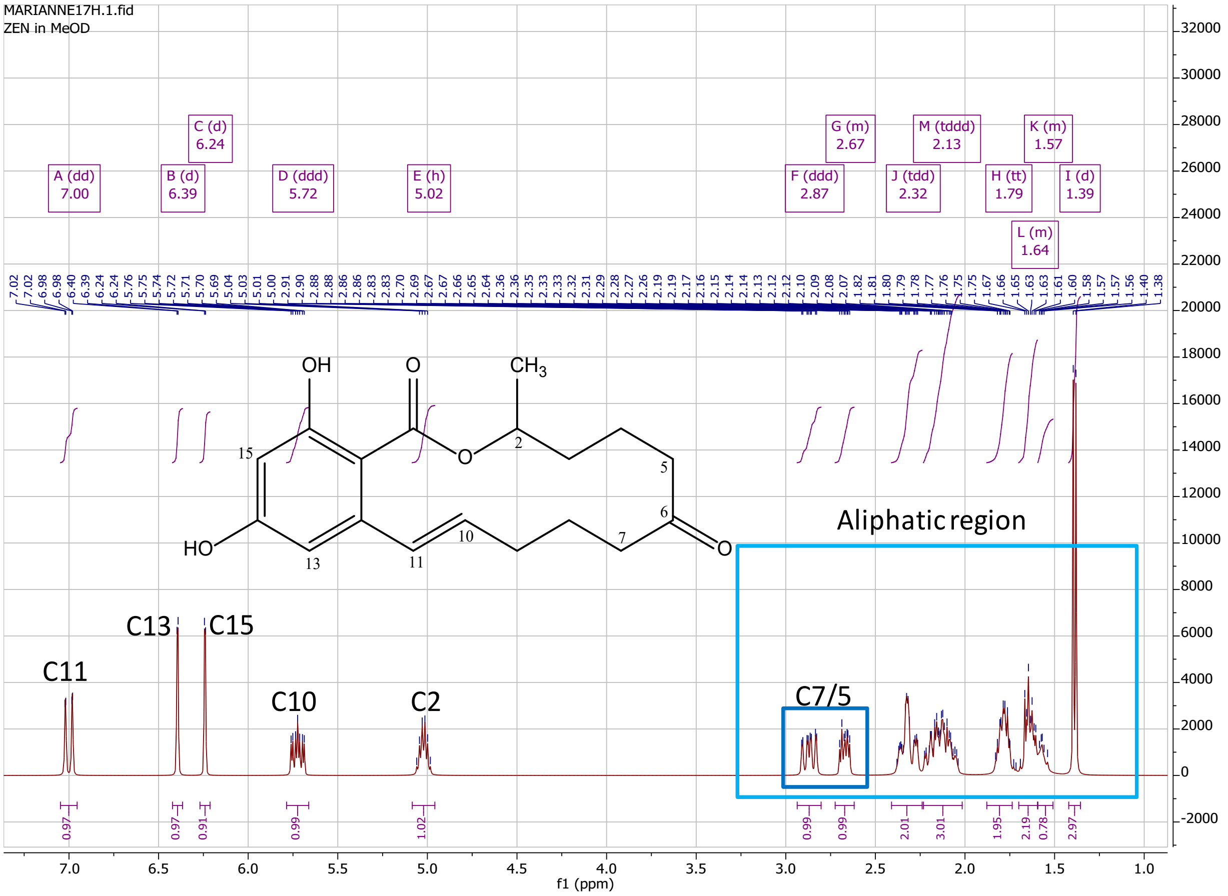

Figure S2. ^{1}H-NMR spectrum of ZEN.

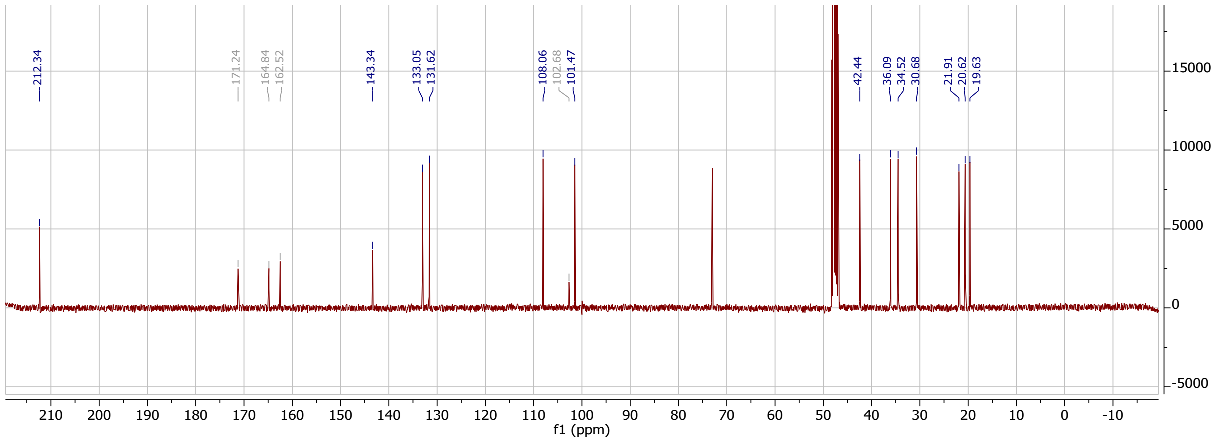

Figure S3. ^{13}C-NMR spectrum of ZEN.

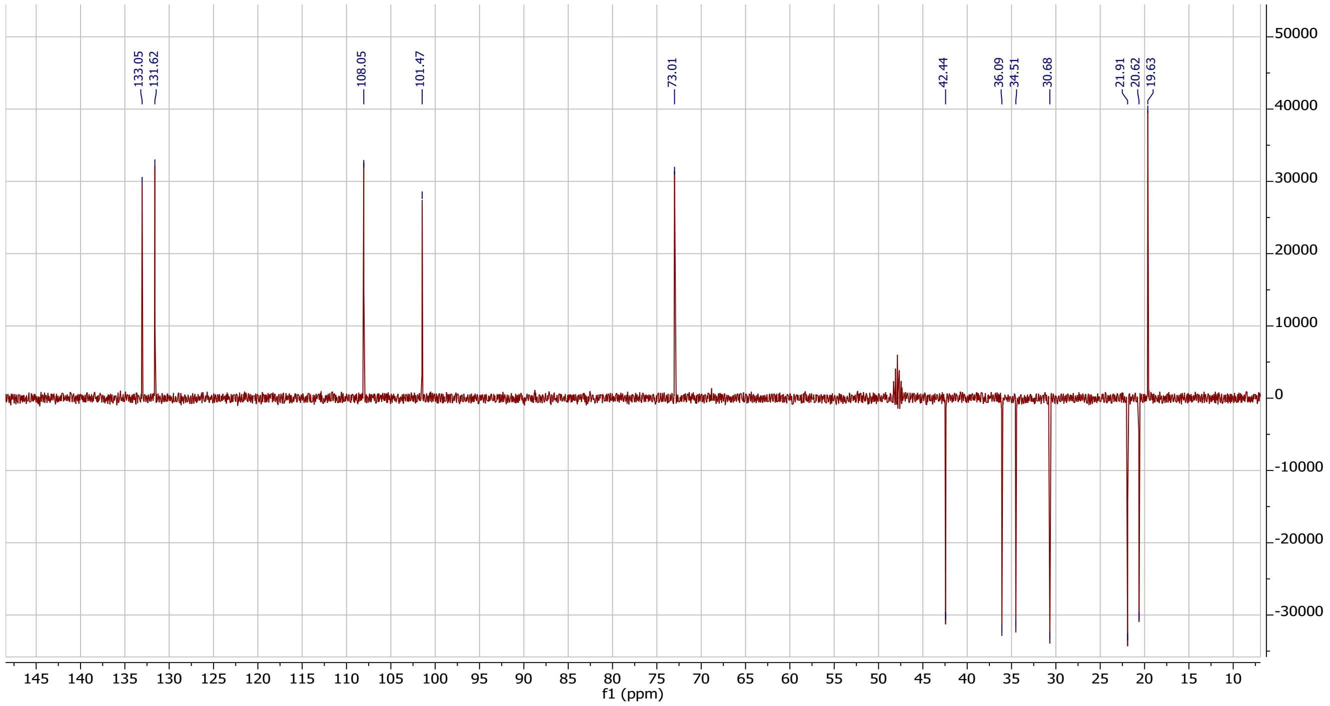

Figure S4. DEPT spectrum of ZEN.

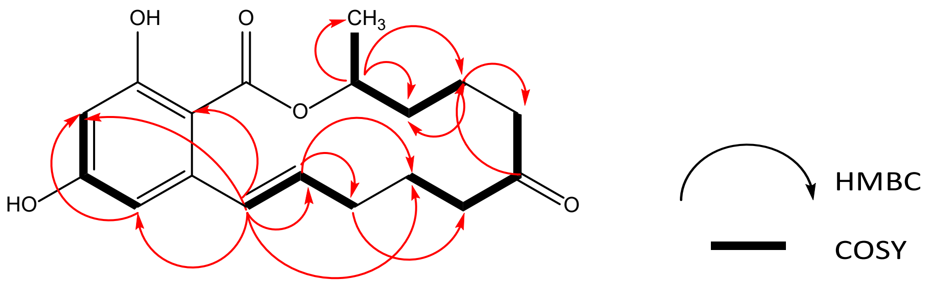

Figure S5. The most important 2D-coupling signals in ZEN.

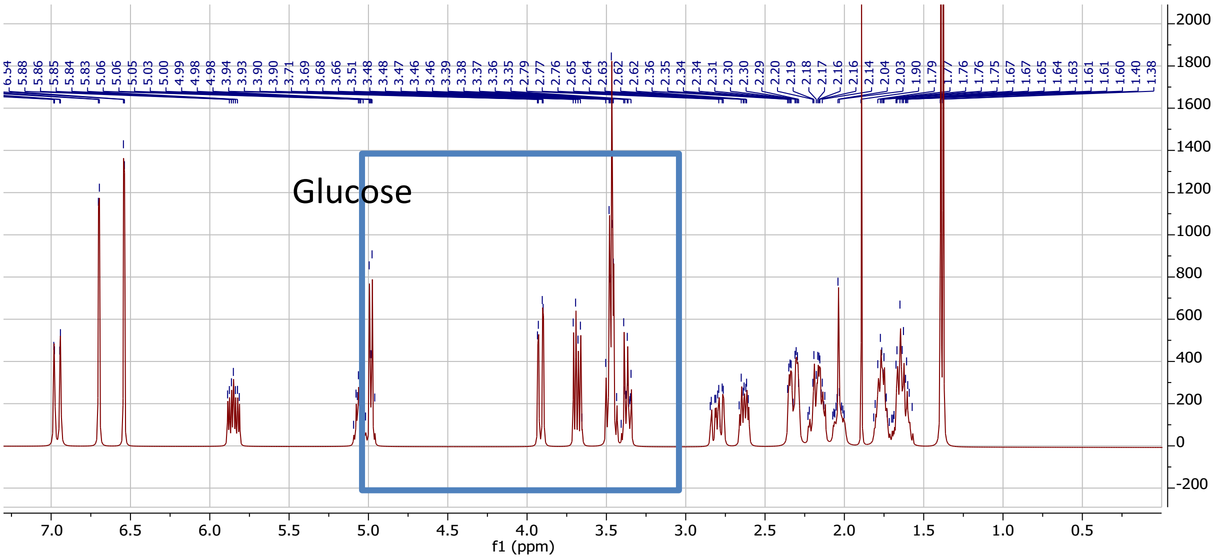

Figure S6. ^{1}H-NMR spectrum of Z14G.

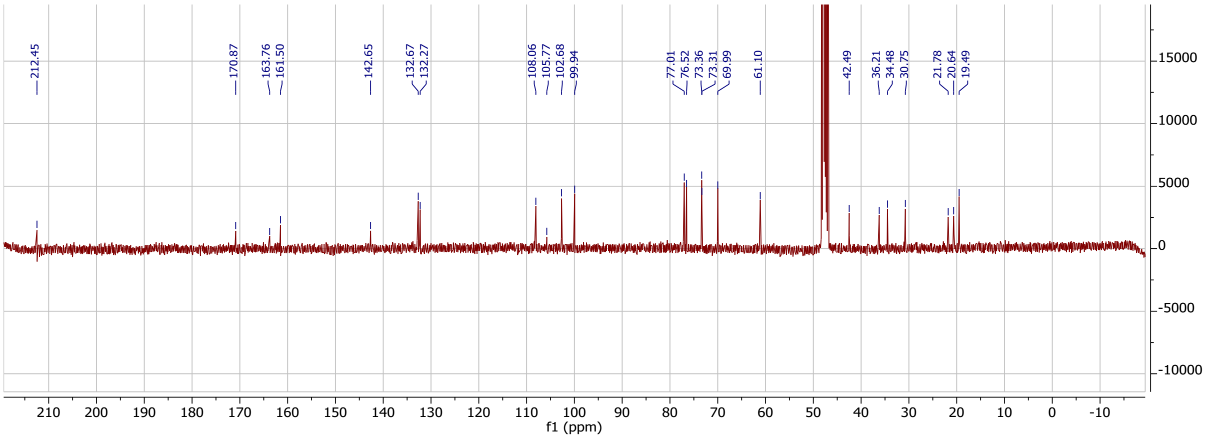

Figure S7. ^{13}C-NMR spectrum of Z14G.

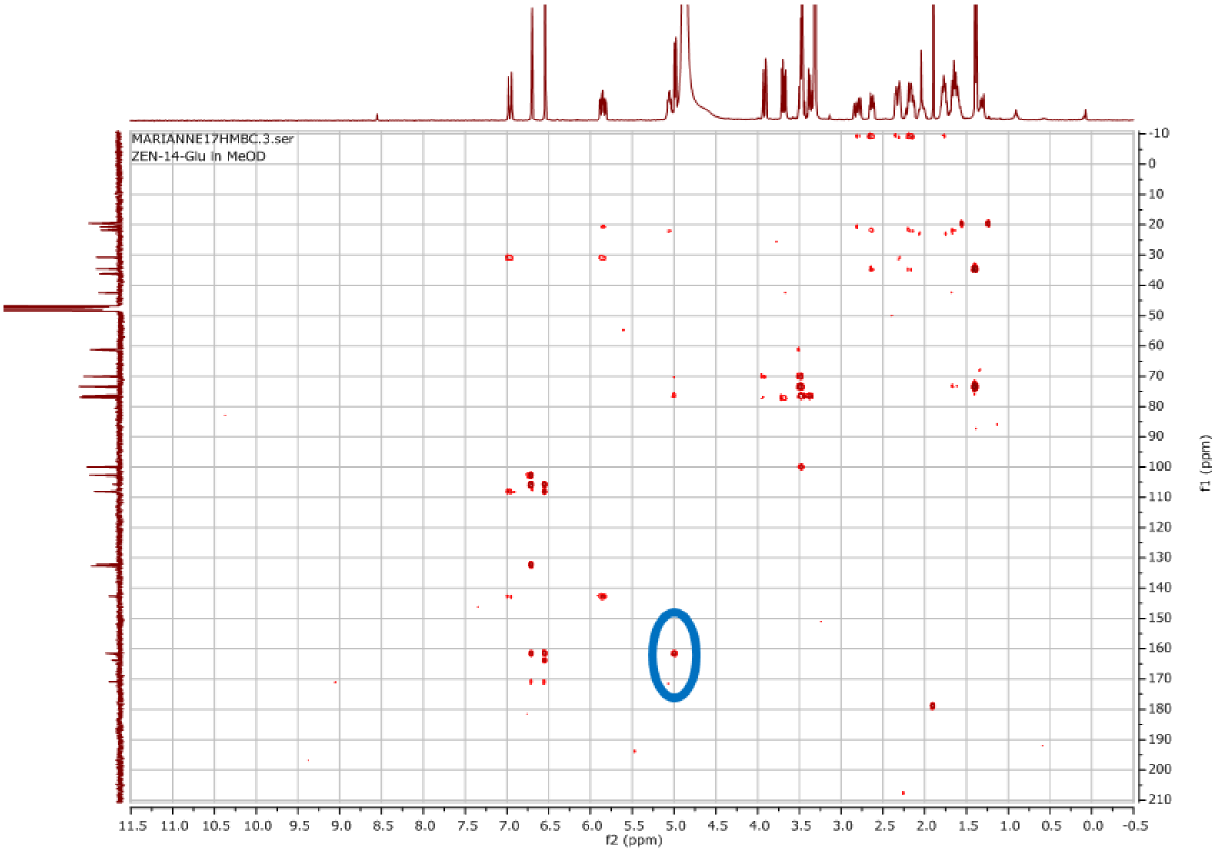

Figure S8. Key HMBC coupling between C14 and C19 in Z14G.

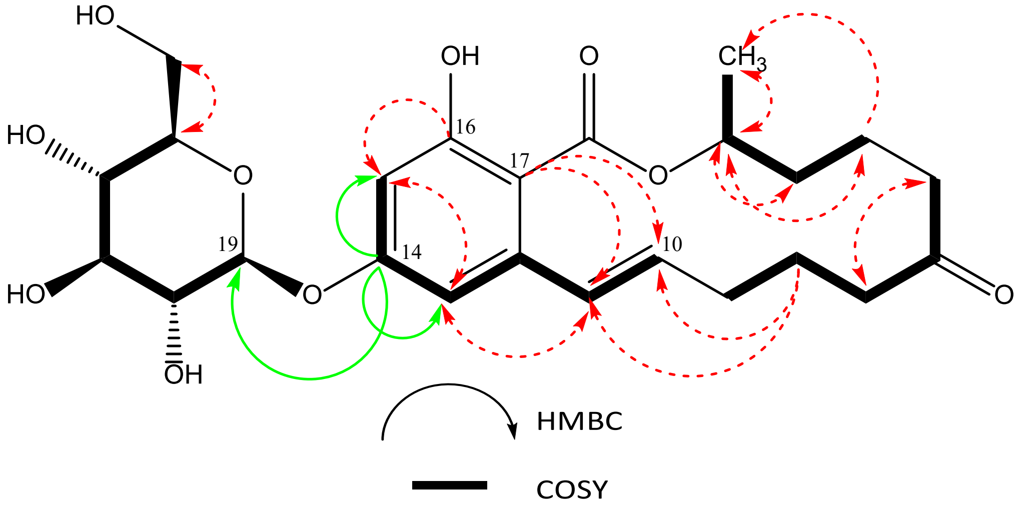

Figure S9. Key HMBC and COSY interactions in Z14G; the green arrows are the couplings that confirm the compound as the C14 conjugate.

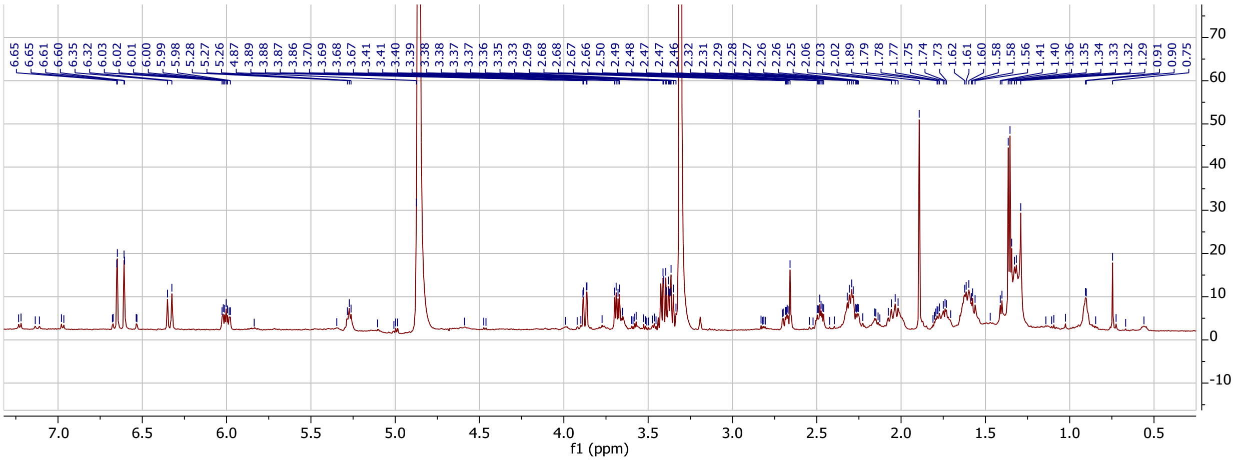

Figure S10. ^{1}H-NMR spectrum of Z16G.

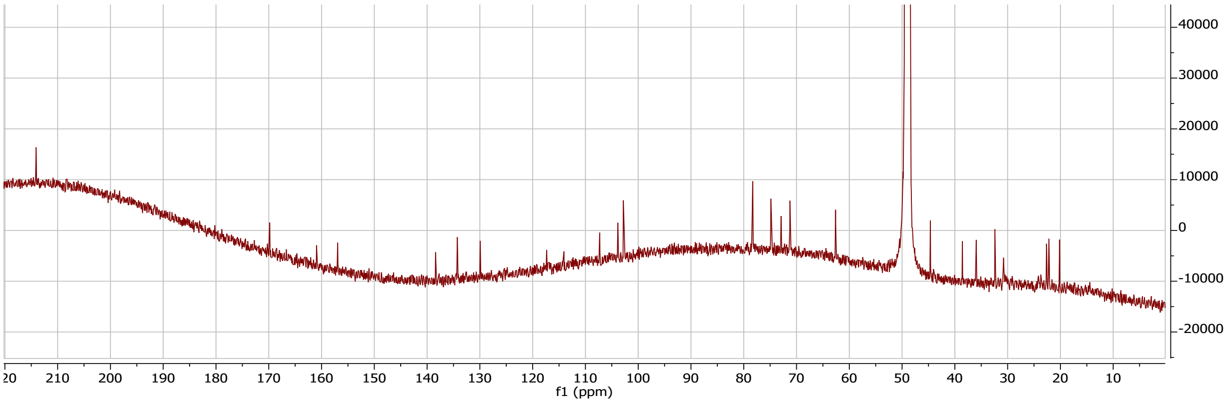

Figure S11. ^{13}C NMR spectrum for Z16G; wavy baseline due to low concentration.

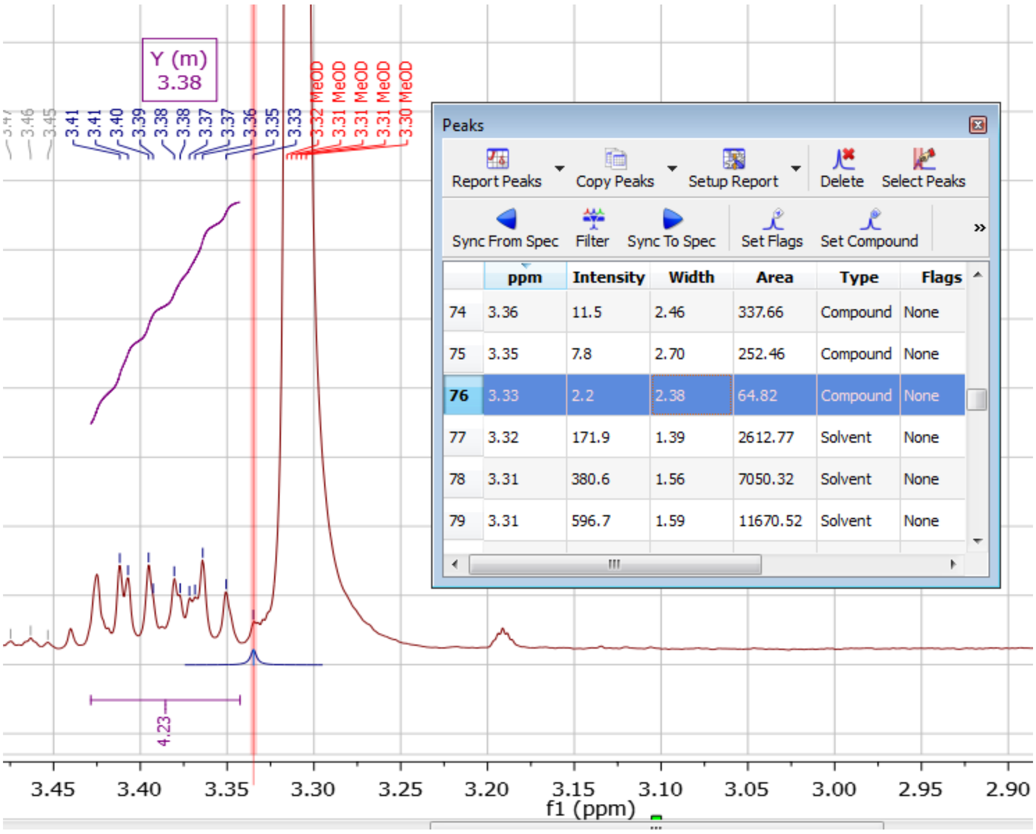

Figure S12. Z16G ^{1}H NMR, showing the shoulder on the MeOD peak indicating the proton on the anomeric C19 which couples to C16—see HMBC in Figure S9.

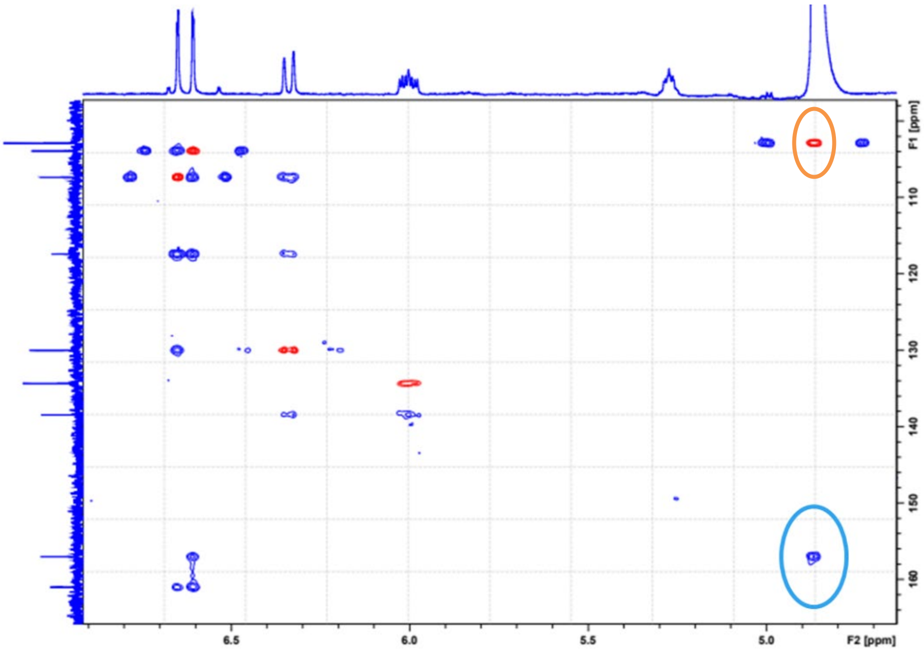

Figure S13. Key HMBC coupling between C16 and C19 in Z16G circled in blue; red couplings are HSQC, blue is HMBC. Circled in green is the HSQC coupling between C19 and its α-hydrogen.

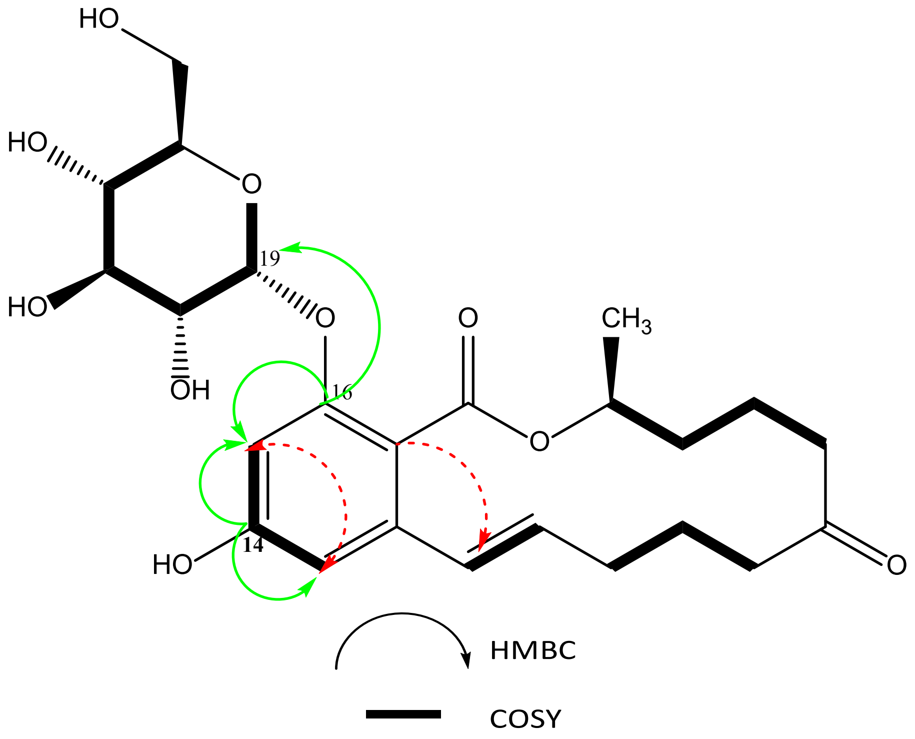

Figure S14. Key HMBC and COSY interactions in Z16G; the green arrows are the couplings that confirm the compound as the C16 conjugate.

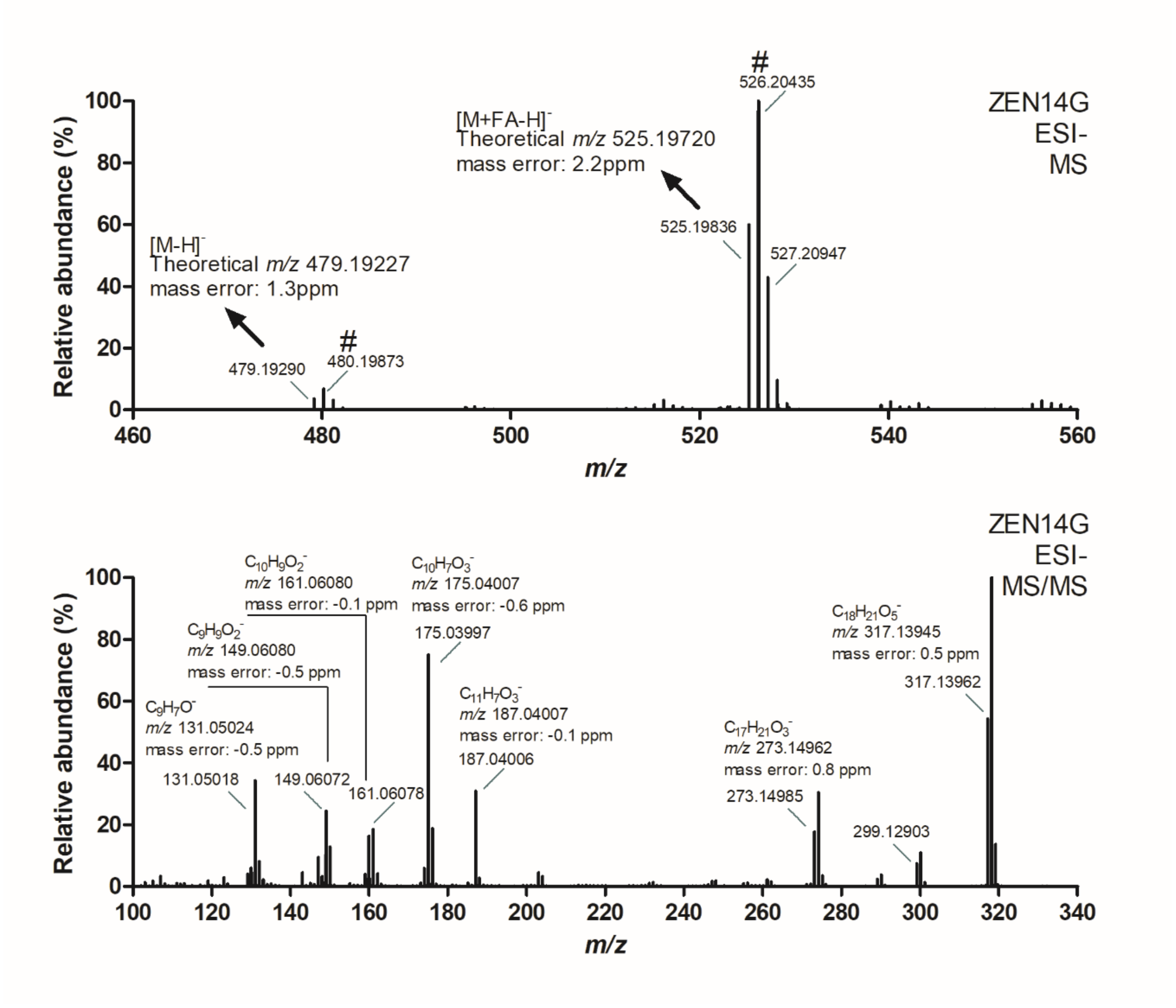

Figure S15. High resolution mass spectra and fragmentation spectra of Z14G (# obtained radical anion).

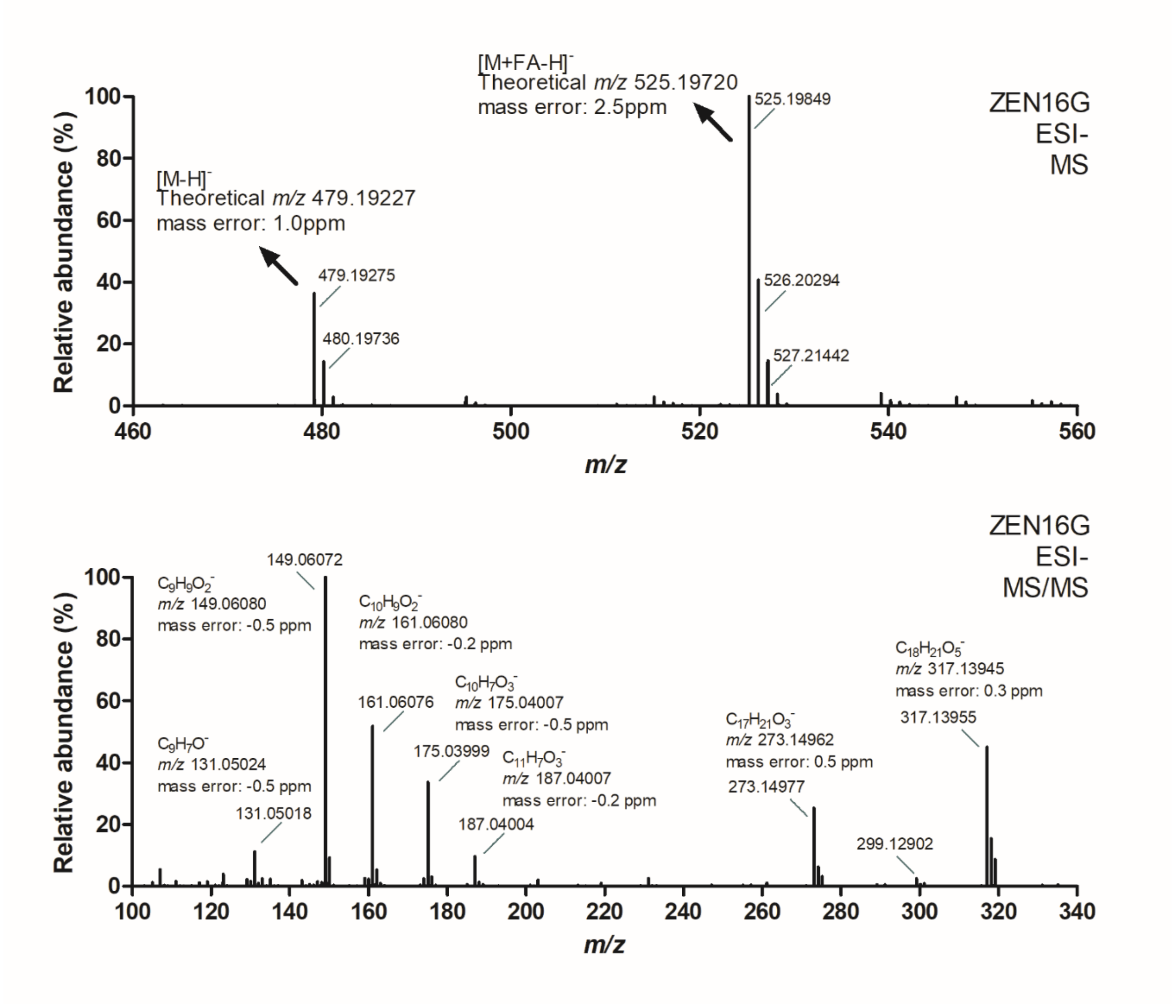

Figure S16. High resolution mass spectra and fragmentation spectra of Z16G.

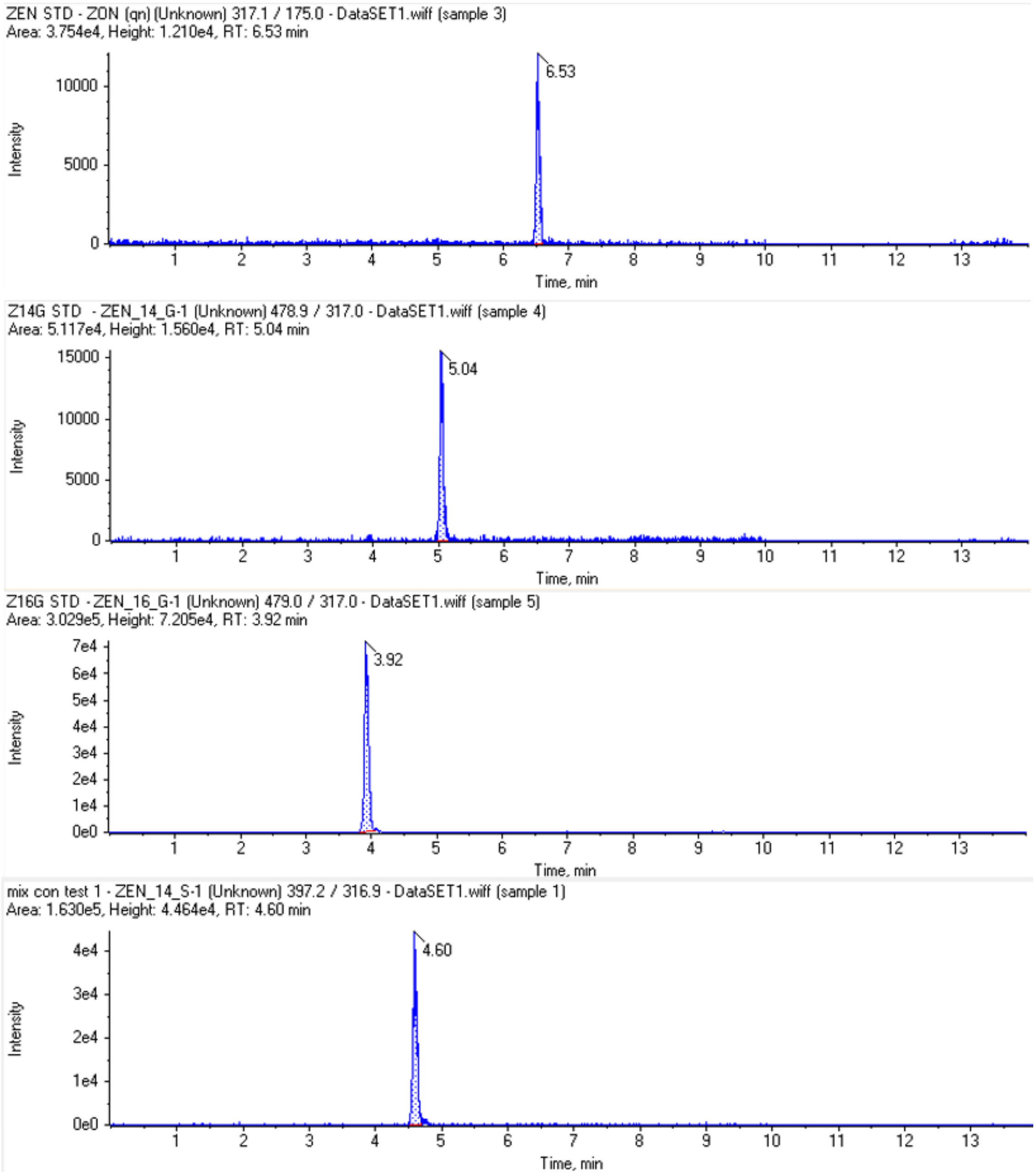

Figure S17. Applied ZEN reference standards in the developed LC-MS/MS method, with (**A**) ZEN, (**B**) Z14G, (**C**) Z16G and (**D**) Z14S.

Table S1. ^{1}H NMR data of ZEN.

^{1}H Peak	ppm	Integral	Class	J1	J2	J3	J4	COSY (^{1}H peak #)	HSQC (^{13}C peak #)	Carbon type	Identity
1	6.99	1	dd	15.3	1.9			2, 3, 4	6	alkene	C11
2	6.38	1	d	2.4				1, 3	8	benzene	C13
3	6.22	1	d	2.5				1, 2	10	benzene	C15
4	5.71	1	ddd	14.8	10.2	4.0		1, 9	7	alkene	C10
5	5.01	1	h	6.1				11, 13	11	ester	C2
6	2.85	1	ddd	18.9	11.9	2.6		7, 8, 9	13	ketone neighbour	C5
7	2.66	1	m					6, 8, 9	12	ketone neighbour	C7
8	2.3	2	tdd	18.7	5.3	2.3		6, 7, 9, 11	13, 15	aliphatic	C5/C8
9	2.12	3	tddd	19.9	14.6	8.7	3.3	6, 7, 8, 10, 11	12, 15	aliphatic	C7/C8/9
10	1.77	2	tt	11.3	5.5			7, 9, 11, 13	14, 16	aliphatic	C3/4
11	1.64	2	m					10, 13	14, 16	aliphatic	C3/4
12	1.55	1	m					8, 9	17	aliphatic	C9
13	1.37	3	d	6.1				10, 11	18	CH3	C1

Table S2. ^{13}C NMR data of ZEN.

^{13}C Peak	ppm	DEPT (#αH)	HSQC (α H peak) (#αH)		HMBC (β/γ H peak)	Carbon Identity
1	212.34	0				C6
2	171.24	0			2, 3, 5	C18
3	164.84	0			3	C16
4	162.52	0			2, 3	C14
5	143.34	0			1, 4	C17
6	133.05	1	1	1	2, 4, 9?	C11
7	131.62	1	4	1	9	C10
8	108.06	1	2	1	1, 3	C13
9	102.68	0			2, 3	C12
10	101.47	1	3	1	2	C15
11	73.01	1	5	1	10, 11, 13	C2
12	42.44	2	7, 9	1+3	10, 11	C7
13	36.09	2	8, 6	2+1	9	C5
14	34.52	2	10, 11	2+2	5, 7, 10, 13	C3/4
15	30.68	2	8, 9	2+3	1, 4, 6, 8, 13	C8
16	21.91	2	10, 11	2+2	5, 7, 9, 11, 13	C3/4
17	20.62	2	9, 12	3+1	4, 6, 8	C9
18	19.63	3	13	3	5, 11, 12	C1

Table S3. ^{1}H-NMR data of Z14G.

Peak	ppm	Integral	Class	J1	J2	J3	COSY (^{1}H peak #)	HSQC (^{13}C peak #)	Carbon type	Identity
1	6.96	1	dd	15	1.7		2, 3, 4	7	alkene	C11
2	6.74	1	d	2.5			1, 3, 4	8	benzene	C13
3	6.54	1	d	2.5			1, 2	10	benzene	C15
4	5.85	1	ddd	15	9.8	4.5	1, 2, 13, 14	6	alkene	C10
5	5.06	1	m				17, 18, 20	15	CH1, ester	C2
6	4.99	2	m				9	11	glucose & OH?	C19 & an OH?
7	3.92	1	dd	12.1	2.2		8, 9	17	CH2 Glc	C24
8	3.69	1	dd	12.1	6.1		7, 9, 10	17	CH2 Glc	C24
9	3.47	3	dd				6, 7, 8, 10	12, 13, 14	glucose	C20 &22 &23
10	3.37	2	m				8, 10	16	glucose and OH?	C21 & an OH?
11	2.81	1	ddd	18.8	11.4	2.7	13, 15	19	ketone	C7
12	2.63	1	m				14, 17	18	ketone	C5
13	2.32	2	ddd	18.9	6.2	2.9	11, 18, 19	19, 21	aliphatic and ketone	C8 & 5
14	2.17	2	m				13, 17, 18, 19	18, 21	aliphatic and ketone	C8 & 7
15	2.03	1	m				11, 18, 19	23	aliphatic	C9
16	1.9	1	s				-	-	OH?	OH?
17	1.77	2	m				5, 12, 14, 18	20, 22	aliphatic	C3 & 4
18	1.65	2	m				5, 13, 14, 15	20	aliphatic	C3 & 4
19	1.6	1	m				13, 14, 15	23	aliphatic	C9
20	1.39	3	d	6.2			5	24	CH3	C1

Table S4. ^{13}C-NMR data of Z14G.

Peak	ppm	DEPT (#αH)	HSQC (α H peak)	(#α H)	HMBC (β/γ H peak)	Carbon Identity
1	212.45	0				C6
2	170.87	0			2, 3	C18
3	163.76	0			3	C16
4	161.5	0			2, 3, 6	C14
5	142.65	0			4, 1	C17
6	132.67	1	4	1		C10
7	132.27	1	1	1	2	C11
8	108.06	1	2	1	1, 3	C13
9	105.77	0			2, 3	C12
10	102.68	1	3	1	2	C15
11	99.94	1	6	2	9	C19
12	77.01	1	9	3	7, 8	C23
13	76.52	1	9	3	6, 9, 10	C20
14	73.36	1	9	3	10	C22
15	73.31	1	5	1	18, 20	C2
16	69.99	1	10	2	6, 7, 9	C21
17	61.1	2	7, 8	1+1	9	C24
18	42.49	2	12, 14	1+2	17, 18	C7
19	36.21	2	11, 13	1+2	20	C5
20	34.48	2	18, 17	2+2	12, 14, 20	C3/4
21	30.75	2	13, 14	2+2	1, 4, 13	C8
22	21.78	2	17	2	5, 12, 14, 18	C3/4
23	20.64	2	15, 19	1+1	5, 11	C9
24	19.49	3	20	3	19	C1

Table S5. ^{1}H-NMR data of Z16G.

Peak	ppm	Integral	Class	J1	J2	J3	COSY (^{1}H peak #)	HSQC (^{13}C peak #)	Carbon type	Identity
1	6.65	1	d	2.1			2	9	benzene	C13
2	6.61	1	d	2.1			1	10	benzene	C15
3	6.34	1	d	15.4			4, 12, 13, 16	7	alkene	C11
4	6.00	1	ddd	4.6	9.8	15.1	1, 2, 12, 13	6	alkene	C10
5	5.27	2	s				13, 16	13	ester	C2
6	4.87	under solvent	n/a				9	11	glucose	C19
7	3.87	1	dd	2.1	12.1		8, 9	16	glucose	C24
8	3.68	2	dd	5.4	12.1		5, 9	15, 16	glucose	C24
9	3.38	4	m				7, 9	12, 13, 15	glucose	C20/21/22/23
10	2.67	2	m				12, 13, 16	18	ketone	C5
11	2.48	1	td	4.6	9.0		12, 15, 16	17	ketone	C7
12	2.29	1	m				10, 11, 13, 15, 16, 17	17, 18	aliphatic	C5/7/9
13	2.05	2	m				10, 12, 16	20, 21	aliphatic	C8/9
14	1.89	2	s						OH?	
15	1.79	2	m				11, 12, 16, 17	19, 22	aliphatic	C3/4
16	1.59	2	m				11, 12, 15, 17	19, 22	aliphatic	C3/4
17	1.34	3	m				12, 15, 16, 18	23	aliphatic	C1
18	0.91	3	s				17		OH?	

Table S6. ^{13}C-NMR data of Z16G.

Peak	ppm	HSQC (α H peak)	(#α H)	HMBC (β/γ H peak)	Carbon Identity
1	214.1		0	11, 12	C6
2	169.8		0	1, 2	C18
3	161.0		0	1, 2	C14
4	156.9		0	2, 6	C16
5	138.4		0	4	C17
6	134.3	4	1		C10 (alkene)
7	129.9	3	1	1, 2	C11 (alkene)
8	117.3		0	1, 2	C12
9	107.3	1	3	2, 3	C13
10	103.9	2	3	1	C15
11	102.8	6	1	9	C19
12	78.3	9	1	6, 7, 8, 9	C20/23
13	74.8	9	1	9, 17	C21
14	72.0	5	1	17	C2
15	71.2	9	1+1	9	C22
16	62.6	7, 8	2	7, 8, 9	C24
17	44.7	11, 12	2	9?	C7
18	38.6	10, 12	2	17?	C5
19	35.9	15, 16	2	11, 17	C3
20	32.4	12, 13	2	3, 4, 9, 15, 17	C9
21	22.7	13	2		C8
22	22.2	15, 16	2		C4
23	20.2	17	3		C1

Table S7. Calculated and HRMS established ions *(m/z)* of ZAN biotransformation metabolites.

Metabolite	Expected position moiety	Calculated [M-H]-	Experimental [M-H]-	LC retention time
Zearalanon	-	319.1551	319.1555	10.65
Zearalanon-sulfate	14 or 16	399.1119	399.1126	9.01
Zearalanon-glucose1	14 or 16	481.2079	481.2082	9.01
Zearalanon-glucose2	14 or 16	481.2079	481.2084	8.20

Table S8. Calculated and HRMS established ions *(m/z)* of β-ZEL biotransformation metabolites.

Metabolite	Expected position moiety	Calculated [M-H]-	Experimental [M-H]-	LC retention time
β-zearalenol	-	319.1551	319.1555	10.06
β-zearalenol-sulfate	7,14 or 16	399.1119	399.1124	8.26
β-zearalenol-14-glucose	14	481.2079	481.2081	8.27
β-zearalenol-glucose1	7 or 16	481.2079	481.2083	7.51
β-zearalenol-glucose2	7 or 16	481.2079	481.2084	9.12

Table S9. LC gradient (LC-MS/MS).

Time (min)	%A	%B
0.0	100	0
1.0	100	0
2.0	50	50
3.0	50	50
8.00	0	100
10.0	0	100
10.5	100	0
15.0	100	0

Table S10. Mycotoxin specific MS/MS settings for the negative ionization mode.

Q1 (*m/z*)	Q3 (*m/z*)	Rt (min)	Analyte	DP (V)	EP (V)	CE (V)	CXP (V)
317.1	175	6.6	ZEN_1	-175	-10	-32	-15
317.1	131.1	6.6	ZEN_2	-175	-10	-36	-11
478.9	317	3.9	Z14G_1	-105	-10	-26	-27
478.9	273	3.9	Z14G_2	-105	-10	-42	-19
479	317	5	Z16G_1	-90	-10	-30	-25
479	273	5	Z16G_2	-90	-10	-44	-21
397.2	316.9	4.5	Z14S_1	-55	-10	-30	-21
397.2	175	4.5	Z14S_2	-55	-10	-46	-11

[1] Q1 = Precursor ion, Q3 = Product ions, Rt = LC retention time, DP = Declustering potential, EP = Entrance Potential, CE = Collision Energy and CXP = Collision cell exit potential

Table S11. LC gradient (LC-HRMS).

Time (min)	%A	%B
0.0	100	0
10.0	0	100
12.0	0	100
12.5	100	0
16.0	100	0
